# Epidemiological and Genetic Characterization of Norovirus Outbreaks That Occurred in Catalonia, Spain, 2017–2019

**DOI:** 10.3390/v14030488

**Published:** 2022-02-27

**Authors:** Eduard Anfruns-Estrada, Sara Sabaté, Efrén Razquin, Thais Cornejo Sánchez, Rosa Bartolomé, Nuria Torner, Conchita Izquierdo, Nuria Soldevila, Lorena Coronas, Àngela Domínguez, Cristina Fuentes, Rosa M. Pintó, Albert Bosch, Susana Guix, The Working Group for the Study of Outbreaks of Acute Gastroenteritis in Catalonia (PI16/02005)

**Affiliations:** 1 Enteric Virus Laboratory, Department of Genetics, Microbiology and Statistics, University of Barcelona, 08028 Barcelona, Spain; eanfruns@ub.edu (E.A.-E.); cfuentes@ub.edu (C.F.); rpinto@ub.edu (R.M.P.); abosch@ub.edu (A.B.); 2 Nutrition and Food Safety Research Institute (INSA·UB), University of Barcelona, 08921 Santa Coloma de Gramenet, Spain; 3 Laboratori de l’Agència de Salut Pública de Barcelona (ASPB), 08023 Barcelona, Spain; ssabate@aspb.cat (S.S.); erazquin@aspb.cat (E.R.); 4 Microbiology Laboratory, Vall d’Hebron University Hospital, 08035 Barcelona, Spain; tcornejosan@gmail.com (T.C.S.); rbartolome@vhebron.net (R.B.); 5 Department of Health, Generalitat of Catalonia, 08005 Barcelona, Spain; nuria.torner@gencat.cat (N.T.); conchita.izquierdo@gencat.cat (C.I.); 6 CIBER de Epidemiología y Salud Pública (CIBERESP), Instituto de Salud Carlos III, 28029 Madrid, Spain; nsoldevila@ub.edu (N.S.); coronas@ub.edu (L.C.); angela.dominguez@ub.edu (À.D.); 7 Department of Medicine, University of Barcelona, 08036 Barcelona, Spain

**Keywords:** human norovirus, acute gastroenteritis outbreaks, molecular epidemiology, Spain, genotyping, viral diversity

## Abstract

Molecular characterization of human norovirus (HuNoV) genotypes enhances the understanding of viral features and illustrates distinctive evolutionary patterns. The aim of our study was to describe the prevalence of the genetic diversity and the epidemiology of the genotypes involved in HuNoV outbreaks in Catalonia (Spain) between 2017 and 2019. A total of 100 HuNoV outbreaks were notified with the predominance of GII (70%), followed by GI (27%) and mixed GI/GII (3%). Seasonality was observed for GII outbreaks only. The most prevalent genotypes identified were GII.4[P31] Sydney 2012, GII.4[P16] Sydney 2012 and GII.2[P16]. As compared to person-to-person (P/P) transmitted outbreaks, foodborne outbreaks showed significantly higher attack rates and lower duration. The average attack rate was higher in youth hostel/campgrounds compared to nursing homes. Only genotypes GI.4[P4], GII.2[P16], GII.4[P16], GII.4[P31] and GII.17[P17] were consistently detected every year, and only abundance of GII.2[P16] showed a negative trend over time. GII.4 Sydney 2012 outbreaks were significantly associated to nursing homes, while GII.2[P16] and GI.3[P3] were most frequently identified in youth hostel/campgrounds. The average attack rate was significantly higher when comparing GII.2[P16] vs. GI.4[P4], GII.2[P16] vs. GII.4[P31] Sydney 2012, and GII.6[P7] vs. GII.4[P31] Sydney 2012. No correlations were found between genotype and outbreak duration or age of affected individuals.

## 1. Introduction

Enteric viruses are the most common cause of acute gastroenteritis. Specifically, human noroviruses (HuNoV) are recognized as the most prevalent agent, causing approximately 17–18% of the total diarrheal diseases and 200,000 annual deaths [1,2]. They also represent the leading cause of gastroenteritis outbreaks worldwide affecting all age groups, being mainly transmitted via an oral-fecal route [3] and with a seasonal pattern, as most of the outbreaks occur within cold months of the year [4].

The genome of HuNoV is organized into three open reading frames (ORFs). ORF1 encodes for non-structural proteins including RNA-dependent RNA polymerase (RdRp), and subsequently ORF2 and ORF3 encode for structural proteins VP1 (major viral protein) and VP2 (minor viral protein), respectively [5]. Recombination between the overlap of ORF1 and ORF2 can occur, and this can lead to the appearance of novel strains containing different RdRp and VP1 combinations [6,7]. The diversity of existing circulating genotypes, including recombinant strains, is represented with a dual typing approach with information from both RdRp and VP1 proteins to allow a better follow-up of HuNoV strain evolution [8].

A classification of HuNoV into 10 genogroups is currently accepted. Genogroup II (GII) accounts for the largest number of infections followed by genogroup I (GI) [9], and, to a much lesser extent, GIV, GVIII and GIX. GI includes 9 VP1 (GI.1-GI.9) and 14 RdRp (GI.P1-GI.P14) different genotypes, and GII includes 26 VP1 (GII.1-GII.14, GII.16-GII.27) and 37 RdRp (GII.P1-GII.P8, GII.P11-GII.P13, GII.P15-GII.P18, GII.P20-GII.P41) genotypes [10].

Within genogroup II, capsid genotype 4 (GII.4) has been the most predominant worldwide for more than the last 20 years [11], with distinct variants emerging periodically every 2–3 years and replacing the previous circulating variant. Variant GII.4 Sydney 2012 emerged in 2012 and has not evolved since then at the capsid antigenic level [8]. Despite this, in 2015, a novel variant of GII.4 Sydney 2012 recombinant with GII.P16 RdRp (GII.4[P16]) appeared in USA [12], suggesting that RdRp mutations may also provide a higher fitness. Since its emergence, GII.4[P16] has been spread widely to many countries including Germany, Canada, Australia, New Zealand and Brazil, resulting in the leading genotype implicated in HuNoV outbreaks in those regions [13,14,15,16]. Conversely, GII.P16 also occurs in association with GII.2 capsid (GII.2[P16]), a genotype that emerged in 2016, and it has also been responsible for the majority of outbreaks during winter 2016–2017 in Germany, Taiwan, Thailand and China [17,18,19,20]. Moreover, during winter 2014–2015, a novel genotype GII.17[P17] emerged in southeast Asia, being implicated in a large number of cases in the area [21] and rapidly extended to other continents [8,22]. However, since 2017, the prevalence of GII.17[P17] appeared to be greatly diminished [8,23,24].

The molecular characterization of the geographical and temporal distribution of a genotype enhances the understanding of viral features, which may affect HuNoV pathology and transmission, and it also illustrates the distinctive evolutionary patterns of HuNoV [25,26]. Before a genotype becomes pandemic, it usually circulates for several years, and, therefore, premature surveillance can help to reduce the overall burden of the disease [24,27].

The main purpose of the study was to describe and analyse the prevalence and the genetic diversity of the different genotypes involved in HuNoV outbreaks between 2017 and 2019 in Catalonia, the second most populated region in Spain with 7.7 million inhabitants. The study also provides information regarding the epidemiology of the declared outbreaks, to test the hypothesis of whether associations between HuNoV genotype and epidemiological characteristics of outbreaks existed.

## 2. Materials and Methods

### 2.1. Sample Collection

Human stool samples were collected from affected individuals during HuNoV outbreaks reported in Catalonia (Spain) through January 2017 to December 2019 in closed and semi-closed settings such as nursing homes, youth/campgrounds, long-term care facilities, schools and other institutions. All epidemiological data were collected by the different Epidemiologic Surveillance Units belonging to the Public Health Agency of Catalonia (ASPCAT). This study was conducted in accordance with the Declaration of Helsinki and was approved by the Ethics Committee of the University of Barcelona (IRB00003099).

### 2.2. HuNoV RTqPCR and Genotyping Assays

The presence of HuNoV in stool was assessed by real-time RTqPCR at the Microbiology Laboratoy at Hospital Universitari Vall d’Hebron and the Agència de Salut Pública de Barcelona (ASPB) [28]. Viral RNA was extracted from a 10% stool suspension using the NucliSENS^®^ easyMAG^®^ system (BioMérieux, Marcy-L’Etoile, France), and the presence of HuNoV was assessed by RTqPCR according to ISO 15216-2:2019 [29].

A semi-nested RT-PCR at the Enteric Virus Laboratory (University of Barcelona) targeting ORF1 and ORF2 genes, including RdRp and VP1, was used for genotyping [8], considering 2–4 positive specimens randomly selected from each outbreak. RT-PCR products were purified and sequenced on an ABI Prism 3700 automatic sequencer (Applied Biosystems, Thermo Fisher Scientific, Waltham, MA, USA). Genotypes were assigned for RdRp and VP1 using the Norovirus Typing Tool (version 2.0) [30]. Phylogenetic analysis was performed using the neighbour-joining method (distance calculation by the Kimura-2-parameter correction; pairwise deletion) implemented in the MEGA7 program [31], and results were validated by 1000 bootstrap replicates.

### 2.3. Statistical Analysis

Chi-square test was used to compare categorical variables by OpenEpi website. Comparisons between means were performed using ANOVA analysis by the Good Calculators website. In addition, *p*-values < 0.05 were considered statistically significant.

## 3. Results

### 3.1. Epidemiological Features of HuNoV Outbreaks and Cases

**Figure 1:**
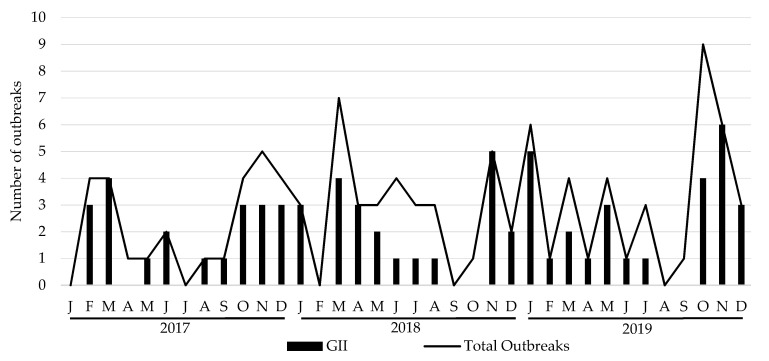
Monthly distribution of total and GII number of HuNoV outbreaks, 2017–2019. J: January; F: February; M: March, A: April; M: May; J: June; J: July; A: August; S: September; O: October; N: November; D: December.

During the study period, a total of 100 HuNoV outbreaks were reported to the Public Health authorities. The total number of outbreaks increased every year, resulting in 27 outbreaks in 2017, 34 in 2018 and 39 in 2019. GII was the predominant genogroup, being involved in 70 outbreaks, followed by GI in 27 and mixed genogroups GI and GII in 3. Monthly distribution of total outbreaks also exhibited a pronounced seasonality with a higher occurrence during cold months (October–March) rather than warm months (April–September) (Figure 1). Information about the outbreak size was reported for 83 of them. Eleven outbreaks affected >50 cases (six in 2017, one in 2018 and three in 2019) and one >250 cases (2017). The main epidemiological features of the studied outbreaks are summarized in Table 1 and Table 2. Average attack rates were significantly higher in foodborne outbreaks as compared to person-to-person (P/P) transmitted outbreaks (45.76% vs. 29.71%, *p* = 0.0106), but duration of outbreak was significantly lower (3.20 vs. 8.93 days, *p* = 0.001). Regarding outbreaks occurring at different settings, differences in mode of transmission were observed between hotels and nursing homes (*p* < 0.003), hotels and schools (*p* = 0.049), and between youth hostel/campgrounds compared with nursing homes (*p* < 0.001) and schools (*p* = 0.0037). Similarly, nursing home outbreaks occurred with a significant higher frequency during cold months, as compared to outbreaks occurring in youth hostel/campgrounds (*p* < 0.001). The average attack rate was significantly higher in youth hostel/campgrounds, as compared to nursing homes (49.22% vs. 28.62%, *p* = 0.013). In addition, a kindergarten/preschool setting showed a significantly longer duration when comparing it with youth hostel/campgrounds (*p* = 0.0059) and hotels (*p* = 0.0077). No significant association was observed between genogroup and mode of transmission. Modes of transmission for GI outbreaks were 77.8% P/P and 22.2% foodborne; for GII outbreaks were 72.9% P/P, 25.7% foodborne and 1.4% waterborne; and for mixed GI/GII outbreaks were 66.7% P/P and 33.3% foodborne.

Symptom information was collected for 533 HuNoV cases (Table 3). Diarrhea presented at a significantly higher frequency in patients older than 65, as compared to patients younger than 15 and to 16–65 age group (*p* < 0.001), and in patients from 16–65 age group compared to patients younger than 15 (*p* < 0.001). Vomiting and fever were significantly less frequent in patients older than 65 years as compared to patients younger than 15 or 16–65 age group (*p* < 0.001). Fever was very rare in patients older than 65.

### 3.2. Prevalence and Evolution of HuNoV Genotypes

Dual genotype information could be obtained from 202 samples of 93 outbreaks. Regarding RdRp, six genotypes were identified combined with GI (P1, P3, P4, P5, P11 and P13), and 10 with GII (P4, P7, P8, P16, P17, P21, P30, P31, P33 and P40). For VP1, six genotypes were identified for GI (GI.1, GI.2, GI.3, GI.4, GI.5 and GI.6) and 10 for GII (GII.1, GII.2, GII.4, GII.5, GII.6, GII.8, GII.10, GII.14 and GII.17). Combined circulating genotypes for GI were GI.1[P1], GI.3 was found to be associated with both P3 and P13 (GI.3[P3] and GI.3[P13]), GI.4[P4], GI.5 with P4 and P5 (GI.5[P4] and GI.5[P5]) and GI.6[P11]. For GII, GII.1[P33], GII.2[P16], GII.3 was associated with P21 and P30 (GII.3[P21] and GII.3[P30]); GII.4 was found to be associated with P4, P16 and P31 (GII.4[P4], GII.4[P16] and GII.4[P31]), GII.5[P40], GII.6[P7], GII.8[P8], GII.10[P16], GII.14[P7] and GII.17[P17].

**Figure 2:**
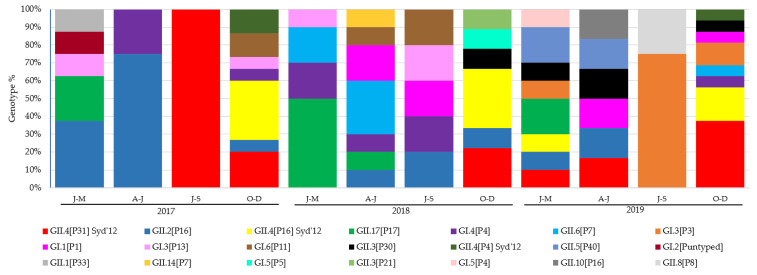
Distribution of genotypes identified per month of 93 typed outbreaks reported during 2017–2019. No outbreaks reported in January, July and August in 2017; February, September in 2018; and August in 2019.

The most prevalent genotypes identified in this study were GII.4[P31] Sydney 2012 being isolated in 14 outbreaks, and each GII.4[P16] Sydney 2012 and GII.2[P16] in 11. For GI, both GI.4[P4] and GI.3[P3] were identified in six outbreaks. Only genotypes GI.4[P4], GII.2[P16], GII.4[P16] Sydney 2012, GII.4[P31] Sydney 2012 and GII.17[P17] were detected all the years of the study period (Table S1). The number and evolution of sequence identification per trimester over the study period are illustrated in Figure 2. 

No strong correlations were observed when analysing epidemiological features according to genotype (Table 4). The average attach rate was significantly higher when comparing GII.2[P16] and GI.4[P4] (55.56% vs. 18.23%, *p* = 0.01), GII.2[P16] and GII.4[P31] Sydney 2012 (55.56% vs. 21.24%, *p* = 0.021), and GII.6[P7] and GII.4[P31] Sydney 2012 (51.24% vs. 21.24%, *p* = 0.047). No other genotype correlations were found with either the duration of the outbreaks or with the mean age of affected individuals. Although not statistically significant, GI outbreaks were less influenced by season as compared to GII outbreaks (*p* = 0.058).

Overall, GII.4 Sydney 2012 outbreaks were significantly associated with nursing homes (*p* = 0.002). Furthermore, GII.2[P16] (*p* = 0.008) and GI.3[P3] (*p* = 0.015) were most frequently identified in youth hostel/campgrounds. 

### 3.3. Phylogenetic Analysis

**Figure 3:**
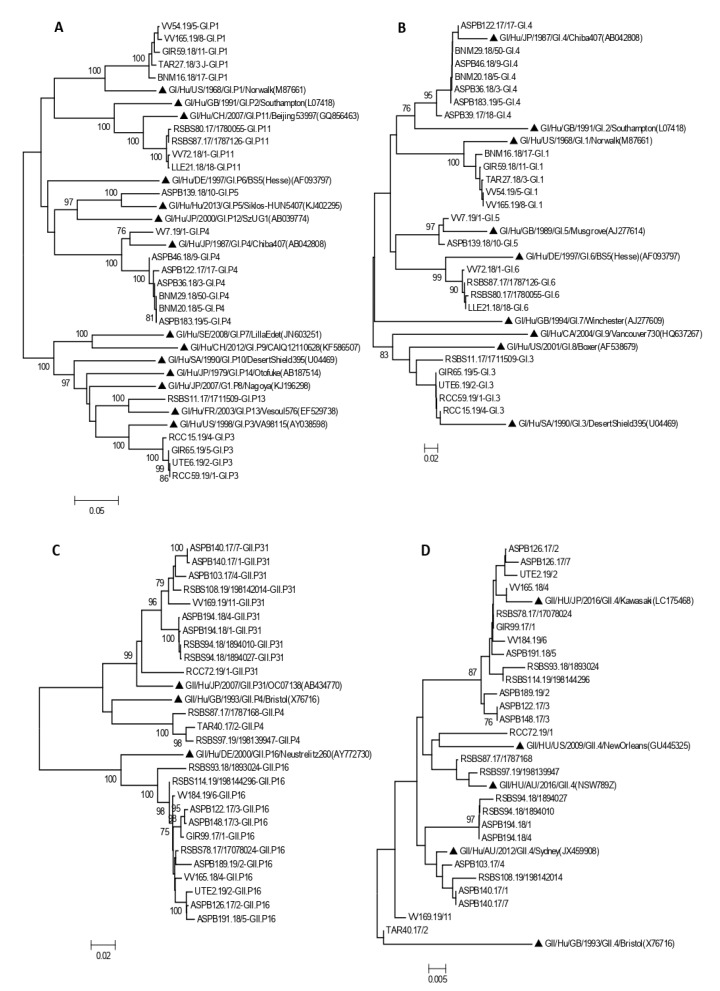

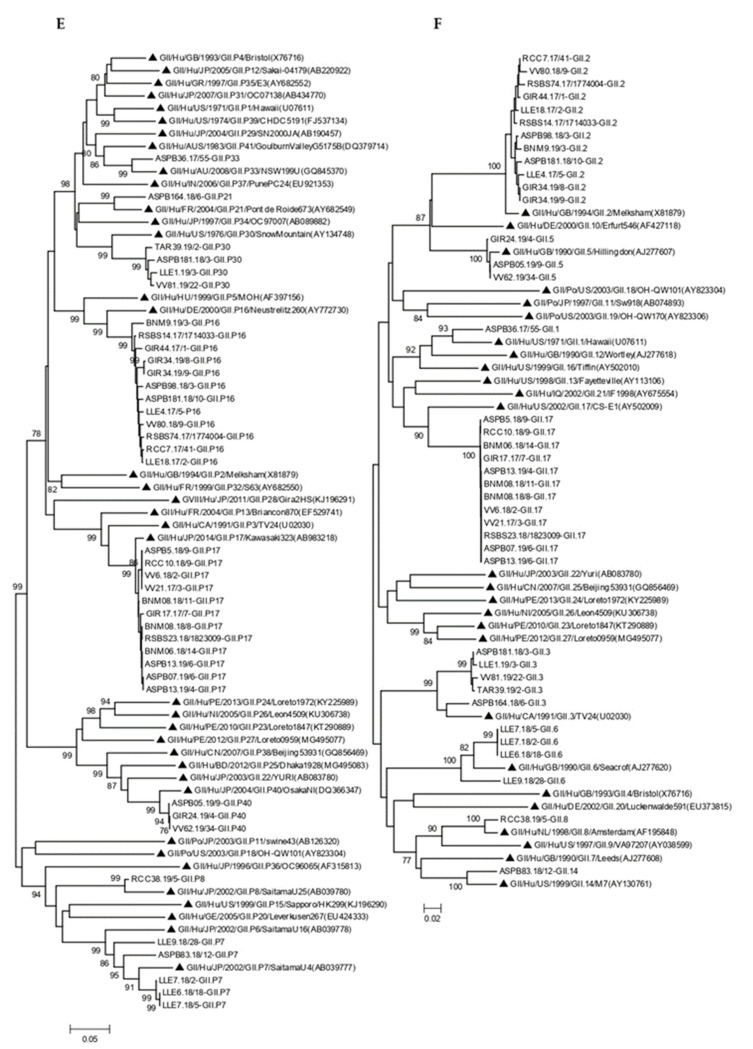
Phylogenetic analysis of HuNoV strains isolated in outbreaks of gastroenteritis, based on RdRp (**A**,**C**,**E**) and VP1 regions (**B**,**D**,**F**). Panels A and B include all GI genotypes, panel C and D include all GII genotypes except GII.4, and panels E and F include all GII.4 variants. Bootstrap values above 75 are shown in the figure. Trees are drawn to scale, with branch lengths in the same units as those of the evolutionary distances used to infer the phylogenetic tree. Symbol indicates reference strains for the respective genotypes. Isolate names are composed of a letter code indicating the geographic region followed by the outbreak number, the year, and sample number.

Phylogenetic analysis confirmed genotype assignment performed using a Norovirus Typing Tool (version 2.0) (Figure 3). Information of 77 of the 93 genotyped outbreaks is shown in the phylogenetic trees.

Four amino acid substitutions not previously described were detected in four samples within the RdRp coding region (Table 5). P1617S (VV165.19/8 GI.P1), L1769F (RSBS87.17/1787126, GI.P11) represented in Figure 3A, L1638R (RSBS74.17/1774004, GII.P16) represented in Figure 3C and P1644T (ASPB140.17/1, GII.P31) represented in Figure 3E.

## 4. Discussion

A total of 100 HuNoV outbreaks were reported during the three-year study, affecting 2677 individuals. In addition, 70% of outbreaks were caused by GII as the leading genogroup, and GII.4 was present in ~40% of them. When compared to a similar study performed in Catalonia during 2010–2012, which identified GII.4 in a higher proportion of outbreaks (66/103) [28], we observed an overall lower proportion of GII.4 outbreaks (including GII.4[P31], GII.4[P4] and GII.4[P16]), and an increase in diversity of other identified HuNoV genotypes over the time (Table S1). These differences could be due to an emerging trend of genotypes other than GII.4, to a lower persistence in the environment or to an absence of immunity against particular genotypes [32].

Despite this lower abundance, GII.4 genotypes were still predominant during the study period. GII.4[P31] Sydney 2012 was the most predominant genotype in Thailand [33] and China [34] during the same period, and along with GII.4[P16] Sydney 2012 were the most identified genotypes in the study, as happened in Germany in 2018 [16]. After GII.4, GII.2[P16] was identified in more than 10% of the outbreaks. This genotype was also the most frequently isolated genotype from sewage in Valencia, Spain in 2016–2017 [35].

As for RdRp genotypes, GII.P16 was the most frequent genotype, isolated in 25% of outbreaks. We detected it in combination with GII.2, GII.4 and GII.10 capsids, although it can also be combined with GII.1, GII.3, GII.13 and GII.12 [24,36]. Due to containing substitutions that enhance RdRp function and virus transmission [37], it is plausible that GII.P16 combined with the fast evolving GII.4 capsids [38] have resulted in a highly transmissible virus [14,15,19].

We identified GII.17[P17] in all three years, mostly in 2018, without observing an increasing trend. Globally, it has been reported that GII.17[P17] reached a peak in 2014/2015, but its incidence began to decline after that [8,39]. A remarkable proportion of GI.3[P3] outbreaks was observed in 2019, being not previously identified in 2017 or 2018, although it did not represent a significant increase in the number of affected individuals that year, as in other genotypes.

Almost one half of the outbreaks occurred in nursing homes, as elderly residents are a highly vulnerable group to the infection who could also experience more severe symptoms. A longer illness duration and extended episodes of excretion have been associated with aged patients, increasing the probability of transmission [40,41,42]. Nevertheless, in our report, a longer duration of outbreaks was significantly associated with kindergarten/preschool setting. Young children are more likely to infect other people possibly because they have wider spheres of activity, lower levels of hygiene and a higher susceptibility to agents due to insufficient acquired immunity [43,44].

Among transmission modes, P/P was identified in 74% of outbreaks as the most common route to acquire HuNoV infection [8,45] followed by foodborne in 24% of them, similar to what has been observed elsewhere [20,46,47]. However, a significantly higher attack rate was observed in foodborne outbreaks, since this mode of transmission is capable of occurring in larger outbreaks affecting a major number of individuals rather than P/P transmission, probably by the ingestion of higher infections doses and the easier identification of individuals exposed to the contaminated source [48,49]. Thus, youth hostel/campgrounds had a significant higher average attack rate over nursing homes because of its mostly identified foodborne transmission origin. Apart from particular food contamination, poor hygienic practices during food preparation can be related to food contamination and the source of foodborne transmission, which could result in a faster virus expansion to consumers [50].

Considered jointly, GII.6[P7] and GII.2[P16] had a higher average attack rate as compared to GII.4[P31] Sydney 2012, probably due to lack of immunity against these less frequent genotypes. In addition, these genotypes were mostly identified in foodborne outbreaks occurring in youth hostel/campgrounds, while GII.4[P31] Sydney 2012 outbreaks occurred predominantly in nursing homes with P/P transmission. An association between GII, particularly GII.4, to P/P transmission and to nursing homes/older patients during cold months has also been reported by previous studies [9,28,51,52]. In our study, ~75% of all three GII.4 Sydney 2012 outbreaks occurred in nursing homes, >85% of the outbreaks occurring during the cold season peak, and ~90% of them were interpersonal outbreaks. Alternatively, in other settings, GI and non-GII.4 are more frequent [53]. In the study, we did observe a significant association of GII.2[P16] and GI.3[P3] with outbreaks occurring in youth hostels or campgrounds and affecting children and young adults. Finally, 68% of the HuNoV outbreaks were reported during cold months (October–March), as people are more frequently clustered indoors, which enhances P/P transmission, and a marked seasonality was especially observed for GII outbreaks only. A lower seasonality observed for GI has also been reported by Matthews et al. [49].

A study of amino acid sequences of partial ORF1 and ORF2 among all typed sequences has been performed to describe novel substitutions. Four amino acid changes have been described in GI.P1, GI.P11, GII.P16 and GII.P31 RdRp sequences compared to those uploaded to GenBank. Point mutations in non-structural proteins could lead to novel properties with better fitness potential in different norovirus genotypes [54].

## 5. Conclusions

Overall, this study shows a great diversity of HuNoV detected as cause of gastroenteritis outbreaks during the three-year period, with 21 different genotypes circulating in the community. Only genotypes GI.4[P4], GII.2[P16], GII.4 [P16], GII.4 [P31] and GII.17[P17] were consistently detected every year, and, of them, only GII.2[P16] showed a reduction in prevalence over time. While a GII.4 Sydney genotype was frequently isolated in outbreaks occurring at nursing homes, outbreaks caused by GII.2[P16] and GI.3[P3] occurred more frequently in youth hostels or campgrounds. Seasonality was strongly observed for GII outbreaks only. Surveillance of HuNoV strains circulating in the community is important for a better understanding of factors driving virus evolution and to provide information for vaccine development.

## Figures and Tables

**Table 1:** Main epidemiological features of outbreaks included in the study, by mode of transmission.

Mode of Transmission	Total Outbreaks	Duration of the Outbreak (Days) ^a^	Total Number of Affected Individuals	Mean of Affected Individuals per Outbreak	Average Attack Rate (%) ^a^
P/P	74	8.93 ± 6.31 ^b^	1795 ^c^	29.42 ± 18.13 ^c^	29.71 ± 20.85 ^c^
Foodborne	22	3.20 ± 2.54 ^d^	696 ^e^	38.66 ± 65.13 ^e^	45.76 ± 26.66 ^e^
Foodborne + P/P	3	4.33 ± 4.16	145	48.33 ± 32	48.12 ± 18.96
Waterborne	1	4	41	41	64

P/P person to person; ^a^ Results are expressed as the mean ± standard deviation ^b^ Information of 59 outbreaks ^c^ Information of 61 outbreaks ^d^ Information of 15 outbreaks ^e^ Information of 18 outbreaks.

**Table 2:** Main epidemiological features of outbreaks included in the study, by setting.

Setting	Total Outbreaks	Mode of Transmission	Season	Duration of the Outbreak (Days) ^a^	Total Number of Affected Individuals	Average Attack Rate (%) ^a^
Nursing home	48	P/P (43), Foodborne (4), Foodborne + P/P (1)	Cold (42), warm (6)	8.86 ± 5.89 ^b^	1208 ^c^	28.62 ± 20.03 ^c^
Youth hostel/Campground	20	Foodborne (12), P/P (5), Foodborne + P/P (2), Waterborne (1)	Warm (12), cold (8)	3.18 ± 2.86 ^d^	537 ^e^	49.22 ± 22.42 ^e^
School	13	P/P (11), Foodborne (2)	Cold (8), warm (5)	9.55 ± 6.25 ^f^	637 ^f^	32.61 ± 23.21 ^f^
Kindergarten/Preschool	6	P/P (6)	Cold (5), warm (1)	12.50 ± 9.09	40 ^g^	23.43 ± 6.16 ^g^
Hotel	6	Foodborne (4), P/P (2)	Cold (3), warm (3)	3.40 ± 3.71 ^h^	113 ^h^	39.12 ± 35.86 ^h^
Long-term care facility	6	P/P (6)	Warm (4), cold (2)	9.33 ± 2.08 ^g^	131 ^i^	35.13 ± 29.17 ^i^
Hospital	1	P/P (1)	Warm (1)	3	11	17

P/P person to person ^a^ Results are expressed as the mean ± standard deviation ^b^ Information of 35 outbreaks ^c^ Information of 41 outbreaks ^d^ Information of 17 outbreaks ^e^ Information of 18 outbreaks ^f^ Information of 11 outbreaks ^g^ Information of 3 outbreaks ^h^ Information of 5 outbreaks ^i^ Information of 4 outbreaks.

**Table 3:** Distribution of cases according to symptoms and age group.

	<15 Years	16–65 Years	>65 Years
Symptom	*n* (%)	*n* (%)	*n* (%)
Diarrhea	123 (61.5)	113 (83.1)	147 (96.7)
Vomiting	196 (91.6)	121 (88.3)	110 (70.1)
Fever	75 (39.9)	53 (47.3)	7 (0.1)

**Table 4:** Main epidemiological features of the outbreaks included in the study, according to genotype.

Genotype	Total Outbreaks	Setting (Number of Outbreaks)	Mode of Transmission	Season	Mean Age of Cases (Years)	Duration of Outbreak (Days)	Total Number of Affected Individuals	Average Attack Rate (%) ^a^
GI.1[P1]	5	Long-term care facility (2), Youth hostel/Campground (2), Hotel (1)	P/P (3), Foodborne (2)	Warm (4), Cold (1)	36.66 ± 20.56	6 ± 5.23 ^b^	107 ^b^	28.35 ± 30.84 ^b^
GI.3[P3]	6	Youth hostel/Campground (4), Nursing home (2)	P/P (3), Foodborne (3)	Warm (3), Cold (3)	46.71 ± 33.12 ^b^	7.67 ± 4.62 ^c^	189	36.93 ± 17.96
GI.3[P13]	2	Youth hostel/Campground (1), School (1)	P/P (1), Foodborne (1)	Cold (1), Warm (1)	19.48 ± 11.72	5.5 ± 3.54	45	48 ± 1.41
GI.4[P4]	6	Nursing home (3), School (2), Hotel (1)	P/P (6)	Cold (3), Warm (3)	43.08 ± 29.89	9.4 ± 8.52 ^b^	115 ^b^	18.23 ± 17.68 ^b^
GI.5[P4]	1	Nursing home (1)	P/P (1)	Cold (1)	62.8	2	18	11.69
GI.5[P5]	1	Nursing home (1)	P/P (1)	Cold (1)	71.31	7	28	17.18
GI.6[P11]	3	Nursing home (2), Youth hostel/Campground (1)	P/P (3)	Warm (2), Cold (1)	52.3 ± 31.74	7 ± 5.29	135	36.34 ± 8.47
Multiple GI genotypes ^k^	1	Nursing home (1)	P/P (1)	Cold (1)	60.72	17	59	83.1
Total GI	25	Nursing home (10), Youth hostel/Campground (8), School (3), Long-term care facility (2), Hotel (2)	P/P (19), Foodborne (6)	Cold (13), Warm (12)	41.85 ± 26.8 ^b^	7.73 ± 6.13 ^d^	721 ^e^	30.71 ± 21.93 ^e^
GII.1[P33]	1	School (1)	P/P (1)	Cold (1)	9.81	9	45	12.47
GII.2[P16]	11	Youth hostel/Campground (6), Nursing home (3), School (1), Hotel (1)	P/P (5), Foodborne (3), Foodborne+ P/P (2), Waterborne (1)	Warm (6), Cold (5)	34.73 ± 25.26	4.82 ± 4.51	434	55.56 ± 24.03
GII.3[P21]	1	School (1)	P/P (1)	Cold (1)	18	ND	ND	ND
GII.3[P30]	3	School (1), Nursing home (1), Hotel (1)	P/P (2), Foodborne (1)	Cold (2), Warm (1)	51.77 ± 27.94	10.5 ± 0.71 ^b^	45 ^b^	42.17 ± 46.43 ^b^
GII.4[P4] Sydney 2012	2	Nursing home (2)	P/P (2)	Cold (2)	94.6 ^b^	12 ^b^	32 ^b^	19.75 ^b^
GII.4[P16] Sydney 2012	11	Nursing home (7) Kindergarten/Preschool (3), Long-term care facility (1)	P/P (11)	Cold (11)	59.38 ± 37.73 ^b^	12.37 ± 8.49 ^c^	255 ^f^	37.52 ± 14.4 ^f^
GII.4[P31] Sydney 2012	14	Nursing home (11), Kindergarten/Preschool (1), Long-term care facility (1), Hotel (1)	P/P (11), Foodborne (3)	Cold (12), Warm (2)	64.02 ± 24.81 ^d^	6.89 ± 6.15 ^d^	206 ^g^	21.24 ± 18.23 ^g^
GII.5[P40]	3	Nursing home (1), Youth hostel/Campground (1), School (1)	Foodborne (2), P/P (1)	Cold (2), Warm (1)	43.15 ± 14.06	4.33 ± 2.31	87	43.36 ± 28
GII.6[P7]	6	Nursing home (2), Youth hostel/Campground (2), School (1), Hotel (1)	P/P (3), Foodborne (2), Foodborne+ P/P (1)	Warm (3), Cold (3)	35.18 ± 22.29	5.33 ± 4.41	153 ^b^	51.24 ± 27.93 ^b^
GII.8[P8]	1	Youth hostel/Campground (1)	Foodborne (1)	Warm (1)	15.6	ND	20	20.41
GII.10[P16]	1	Socio-health centre (1)	P/P (1)	Warm (1)	62	10	32	17.58
GII.14[P7]	1	School (1)	P/P (1)	Warm (1)	18.69	ND	11	42.31
GII.17[P17]	9	Nursing home (6), Youth hostel/Campground (1), School (1) Long-term care facility (1)	P/P (7), Foodborne (2)	Cold (8), Warm (1)	58.89 ± 24.75	6.29 ± 3.55 ^e^	466	32.06 ± 20.8
Multiple GII genotypes ^l^	1	School (1)	P/P (1)	Cold (1)	18.82	12	55	11.58
Total GII	65	Nursing home (33), Youth hostel/Campground (11), School (9), Kindergarten/Preschool (4), Hotel (4), Long-term care facility (3), Hospital (1)	P/P (47), Foodborne (14), Foodborne + P/P (3), Waterborne (1)	Cold (48), Warm (17)	48 ± 31.05 ^h^	7.56 ± 6.22 ^i^	1905 ^j^	37.23 ± 23.4 ^j^
Multiple GI + GII genotypes ^m^	3	Nursing home (2), Kindergarten/Preschool (1)	P/P (2), Foodborne (1)	Cold (3)	49.46 ± 37.1	7 ± 5.66 ^b^	51	13.17 ± 11.26
Total	93	Nursing home (45), Youth hostel/Campground (19), School (12), Kindergarten/Preschool (5), Hotel (6), Long-term care facility (5), Hospital (1)	P/P (68), Foodborne (21), Foodborne + P/P (3), Waterborne (1)	Cold (64), Warm (29)	46.31 ± 30.03	7.59 ± 6.12	2677	34.22 ± 23.14

P/P person to person. ND: not determined. ^a^ Results are expressed as the mean ± the standard deviation. ^b^ Missing information for 1 outbreak. ^c^ Missing information for 3 outbreaks. ^d^ Missing information for 5 outbreaks. ^e^ Missing information for 2 outbreaks. ^f^ Missing information for 4 outbreaks. ^g^ Missing information for 6 outbreaks. ^h^ Missing information for 7 outbreaks. ^i^ Missing information for 12 outbreaks. ^j^ Missing information for 13 outbreaks. ^k^ GI.3[P13]/GI.2[Puntyped]. ^l^ GII.2[P16]/GII.3[P30]. ^m^ GII.4[P16] Sydney 2012/GI.4[P4], GII.17[P17]/GI.3[P13] and GII.4[P4] Sydney 2012/GI.6[P11].

**Table 5:** Location of amino acid changes in RNA-dependent RNA polymerase (RdRp) sequences. Aminoacid positions refer to the corresponding reference strain in each case.

	1608	1609	1610	1611	1612	1613	1614	1615	1616	1617	1618	1619	1620	1621	1622	1623	1624	1625	1626
GI.P1(QCT04921)	E	R	Q	I	F	W	T	R	G	P	N	H	S	D	P	S	E	T	L
VV165.19	E	R	Q	I	F	W	T	R	G	S	N	H	S	D	P	S	E	T	L
	1760	1761	1762	1763	1764	1765	1766	1767	1768	1769	1770	1771	1772	1773	1774	1775	1776	1777	1778
GI.P11(QJC14597)	M	F	R	W	M	R	F	H	D	L	G	L	W	T	G	D	R	N	L
RSBS87.17	M	F	R	W	M	R	F	H	D	F	G	L	W	T	G	D	R	N	L
	1629	1630	1631	1632	1633	1634	1635	1636	1637	1638	1639	1640	1641	1642	1643	1644	1645	1646	1647
GII.P16 (QCE20812)	L	M	A	L	L	G	E	A	S	L	H	G	P	S	F	Y	S	K	I
RSBS74.17	L	M	A	L	L	G	E	A	S	R	H	G	P	S	F	Y	S	K	I
	1635	1636	1637	1638	1639	1640	1641	1642	1643	1644	1645	1646	1647	1648	1649	1650	1651	1652	1653
GII.P31(QCQ77492)	I	V	S	T	D	I	K	L	D	P	E	K	L	T	A	K	L	K	E
ASPB140.17	I	V	S	T	D	I	K	L	D	T	E	R	L	T	A	K	L	K	E

Letters in bold indicate aminoacid changues. Proline (P), Serine (S), Leucine (L), Phenylalanine (F), Arginine (R) and Threonine (T).

## Data Availability

Identified sequences were submitted to GenBank (accession No. OM182806-OM182828 for GI, OM185329-OM185367 for GII and OM185492-OM185517 for GII.4).

## Supplementary Materials

The following supporting information can be downloaded at: https://www.mdpi.com/article/10.3390/v14030488/s1, Table S1: Prevalence of genotypes identified during the study period per year.
