# Supplementary material for: Epidemiological and Genetic Characterization of Norovirus Outbreaks That Occurred in Catalonia, Spain, 2017–2019
Source: Viruses. 2022 Feb 27;14(3):488. doi: 10.3390/v14030488 (PMC8955687; doi:10.3390/v14030488)
Supplement: Supplementary file 1 [file viruses-14-00488-s001.zip › viruses-1595564-supplementary.pdf]

viruses

MDPI

Supplementary Materials

Epidemiological and Genetic Characterization of Norovirus Outbreaks That Occurred in Catalonia, Spain, 2017–2019

Table S1. Prevalence of genotypes identified during the study period per year.

Genotype	*N.* of Outbreaks	2017 (%)	2018 (%)	2019 (%)
GI.1[P1]	5	0 (0%)	3 (8.82)	2 (5.13)
GI.3[P3]	6	0 (0%)	0 (0%)	6 (15.38)
GI.3[P13]	2	1 (3.7)	1 (2.94)	0 (0%)
GI.4[P4]	6	1 (3.7)	4 (11.76)	1 (2.56)
GI.5[P4]	1	0 (0%)	0 (0%)	1 (2.56)
GI.5[P5]	1	0 (0%)	1 (2.94)	0 (0%)
GI.6[P11]	3	1 (3.7)	2 (5.88)	0 (0%)
GII.1[P33]	1	1 (3.7)	0 (0%)	0 (0%)
Multiple GI genotypes [a]	1	1 (3.7)	0 (0%)	0 (0%)
GII.2[P16]	11	7 (25.93)	2 (5.88)	2 (5.13)
GII.3[P21]	1	0 (0%)	1 (2.94)	0 (0%)
GII.3[P30]	3	0 (0%)	0 (0%)	3 (7.69)
GII.4[P4] Sydney 2012	2	1 (3.7)	0 (0%)	1 (2.56)
GII.4[P16] Sydney 2012	11	4 (14.81)	3 (8.82)	4 (10.26)
GII.4[P31] Sydney 2012	14	4 (14.81)	2 (5.88)	8 (20.51)
GII.5[P40]	3	0 (0%)	0 (0%)	3 (7.69)
GII.6[P7]	6	0 (0%)	5 (14.71)	1 (2.56)
GII.8[P8]	1	0 (0%)	0 (0%)	1 (2.56)
GII.10[P16]	1	0 (0%)	0 (0%)	1 (2.56)
GII.14[P7]	1	0 (0%)	1 (2.94)	0 (0%)
GII.17[P17]	9	2 (7.41)	5 (14.71)	2 (5.13)
Multiple GII genotypes [b]	1	0 (0%)	1 (2.94)	0 (0%)
Multiple GI + GII genotypes [c]	3	2 (7.41)	1 (2.94)	0 (0%)
Untyped	7	2 (7.41)	2 (5.88)	3 (7.69)
Total	100	27	34	39

[a] GI.3[P13]/GI.2[Puntyped]; [b] GII.2[P16]/GII.3[P30]; [c] GII.4[P16] Sydney 2012/GI.4[P4], GII.17[P17]/GI3[P13] and GII.4[P4] Sydney 2012/GI.6[P11].
